# Antlion larvae follow optimality rules in body orientation during sand tossing

**DOI:** 10.1242/jeb.252138

**Published:** 2026-05-13

**Authors:** Francesca Lo Bello, Massimo De Agrò, Giorgio Vallortigara

**Affiliations:** Centre for Mind/Brain Sciences, University of Trento, 38068 Rovereto, Italy

**Keywords:** Pit-trap, Sit-and-wait predator, Invertebrate, Vibration

## Abstract

Pit-building antlion larvae (*Myrmeleon* spp.) throw sand at prey that approach their pit using their head and mandibles as a backward-directed ‘biological catapult’. This mechanism restricts effective sand tossing to a defined angular range behind the larva. When prey-like vibrations come from outside this range, antlions are known to reorient, but the consistency and rules behind this behaviour remain unclear. We tested whether larvae follow optimal turning strategies when reorienting toward a vibrating stimulus placed in disadvantageous positions along the pit. Across 128 trials (19 individuals), larvae responded in 45 trials (∼35%). In responding trials, initial orientation strongly predicted turning direction: larvae rotated along the shortest angular path to reach the optimal sand-throwing orientation. These results provide evidence that antlion reorientation towards the prey is non-random and follows a minimisation rule, revealing a previously unnoticed behaviour.

## Teaser

**Summary:** In response to prey-like vibrations, antlion larvae rotate their bodies along the shortest angular path, minimising spatial displacement to reach the optimal sand-throwing position.

## INTRODUCTION

Throughout evolution, natural selection is expected to favour behavioural strategies that maximise efficiency by minimising energetic costs relative to their benefits. In many behavioural contexts, this can result in animals following simple ‘optimality rules’, whereby movements or actions are organised so as to balance speed, safety and energy demands (Pontzer, 2025; Norberg, 2021). These principles may be particularly relevant in the context of predation, where efficient behavioural responses can be crucial for successful prey capture.

Among the various hunting strategies adopted by predators, the widely used ‘sit-and-wait’ approach (ambush strategy) involves remaining immobile until prey approaches and therefore relies on precise and energetically efficient responses. Among insects, antlions (*Myrmeleon* spp., order Neuroptera) represent a successful example of this predatory strategy (for reviews, see Mencinger-Vračko and Devetak, 2008; Scharf and Ovadia, 2006).

During their larval stage, antlions inhabit sandy or loose substrates in which they excavate an inverted conical pit. To do so, they move backward in progressively smaller circles (Coelho, 2001), using their head and mandibles to dig the sand, ultimately ending at the centre of the pit (Coelho, 2001; Scharf et al., 2011). The pit's structure makes it an effective trap because its sloped walls are close to the angle of repose (Lucas, 1982), which depends on the grain size (Franks et al., 2019; Botz et al., 2003): steep enough to prevent prey from escaping, yet stable enough to avoid unwanted sand collapses (Büsse et al., 2021; Fertin and Casas, 2006). Once the pit is completed, the larvae position themselves at the bottom, buried beneath the sand with only the antennae and the anterior edges of the mandibles exposed (Coelho, 2001; Griffiths, 1980; Lucas, 1989; Hollis et al., 2011). When a prey falls into the pit, the steep and unstable walls cause the prey to slide toward the centre where the antlion larvae, positioned under the sand, capture it by closing their jaws (Mencinger-Vračko and Devetak, 2008; Griffiths, 1980; Lucas, 1989).

However, the prey may resist or delay its fall to the bottom of the pit. When this happens, antlions adopt a more active behavioural strategy (Fertin and Casas, 2007): as soon as the prey is positioned roughly behind it, the predator buries its long, curved mandibles into the substrate to collect sand grains and throw them backward toward the prey (Griffiths, 1980; Napolitano, 1998), effectively using its head and mandibles as a ‘biological catapult’ (Mencinger-Vračko and Devetak, 2008; Griffiths, 1980; Fertin and Casas, 2007). This sand-throwing behaviour typically induces small sand slides that both distract the prey and reduce its grip on the pit's surface, ultimately causing it to slip toward the centre, where it is captured by the antlion (Büsse et al., 2021).

To detect approaching prey while remaining buried at the bottom of the pit, antlion larvae rely mainly on substrate-borne vibrational cues generated by prey movement on the sand (Mencinger-Vračko and Devetak, 2008; Fertin and Casas, 2007). They can detect the presence of prey from about 3 cm up to over 12 cm away, depending strongly on sand type, vibration strength and individual sensitivity (Devetak, 2014; Devetak et al., 2007a). They can detect not only the presence but, crucially, the location of the approaching prey by perceiving low-frequency vibrations (Podlesnik et al., 2019) generated by the prey movements on the substrate (Fertin and Casas, 2006; Miler et al., 2018; Miler and Czarnoleski, 2022), propagating through the sand reaching the buried larva (Devetak et al., 2007b). While directional localisation appears to be crucial for effective sand tossing, there is currently no evidence that antlion larvae require an estimation of prey distance. Because sand tossing can affect prey located at different distances within the pit, it could be that orientation relative to the prey may be more important than an exact distance estimate. Antlions are capable of detecting extremely weak vibrations, down to sub-nanometre amplitudes, across a frequency range of approximately 0.1–4.5 kHz (Büsse et al., 2021; Devetak et al., 2007a; Martinez et al., 2020). Vibrational cues are received mainly by mechanoreceptive hairs (sensilla) on the thorax and mouthparts (Acevedo Ramos et al., 2020; Param et al., 2022), which act as the primary vibration receptors involved in prey localisation (Fertin and Casas, 2007; Devetak et al., 2007b). Overall, antlions show very precise directional localisation of prey based on vibration cues (Mencinger-Vračko and Devetak, 2008; Hollis et al., 2011; Fertin and Casas, 2007; Martinez et al., 2023).

Experiments using artificial vibrational pulses showed that the antlions' sand-tossing behaviour is also directional and precise. In fact, antlions direct their sand-tossing attempts towards the side where the artificial prey is located, also suggesting that localisation relies primarily on the onset time difference between the left and right receptors (Devetak, 2014; Martinez et al., 2023). However, it is crucial to point out that the sand-tossing behaviour remains mechanically constrained. Antlions can only toss sand toward the rear of their body, as their throwing mechanism is restricted to a backward arc. This range reflects the limited degrees of freedom of the head–prothorax joint, which makes it mechanically impossible for the larva to project sand toward the anterior sector (Mencinger-Vračko and Devetak, 2008).

While larvae often keep a fixed orientation once pit construction has been completed (Farji-Brener et al., 2025), Devetak (2014) reported that when antlions are facing frontally toward the prey, or after repeated failed sand tosses, they reorient their bodies, sometimes moving closer in order to gain a more advantageous orientation. However, this observation was only qualitative, so it remains unclear how common, flexible or precise this reorientation behaviour is.

The reorientation of the antlion exhibits an intrinsic level of flexibility. The animal in fact may rotate its body either clockwise or counterclockwise within the pit. Depending on the prey location, one of these direction is shorter than the other, which should be preferred in terms of behavioural optimality.

Here, we designed an experiment to investigate the reorientation behaviour of antlions towards artificial prey, located in disadvantageous positions for sand tossing. We hypothesised that antlions will consistently reorient their body upon prey detection and that they will do so following a movement-minimisation rule, preferentially rotating along the shortest angular path to reach the effective sand-tossing orientation.

## MATERIALS AND METHODS

A total of 19 larvae of *Myrmeleon* spp., commonly referred to as antlion, were collected from the wild in the Trentino region (north-east of Italy) during the end of summer 2024. Once in the lab, each animal was individually housed in a square wooden box measuring 8×8×4 cm. Each box was filled to three-quarters of its size with dry sand with a grain diameter of approximately 1–2 mm. Larvae were then left for at least 3 days, to allow them enough time to construct their pitfall traps. The boxes were placed in a room with access to natural light through a window to maintain the natural day/night cycle.

### Experimental procedure

After ensuring the antlion had properly constructed its cone, a video camera Flir GigE (resolution 1280×720, 30 frames s^−1^) was placed 40 cm above the box. An LED lamp was placed above the pit to provide light for the recording. In order to simulate a prey-induced vibration on the sand, three PVC bristles were permanently attached to the posterior edge of a vibrating rod (Satisfyer Dual Love Connect App), allowing vibrations to be transmitted from the device to the bristles. The bristles were then put in contact with the sand substrate, inducing vibration across it from a punctiform starting location.

The device did not allow us to specify the exact frequency or amplitude of the vibration. Nonetheless, this was set empirically, modulating the level until reaching the minimum intensity for which the contact of the PVC bristles with the sand was sufficient to elicit the sand-tossing reaction, but not strong enough to disrupt the sand pit. Once set, the found intensity was then kept as standard for all the trials.

**Fig. 1.**
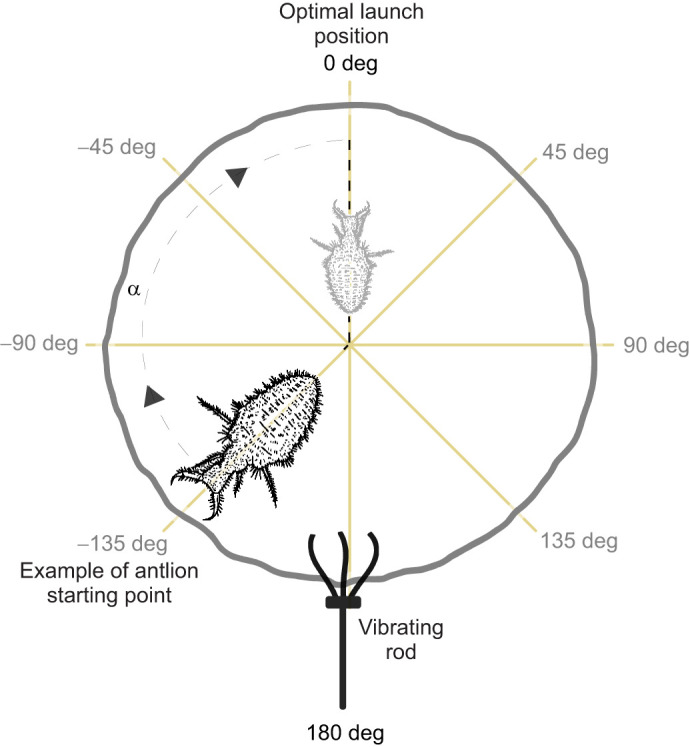
**Experimental setup.** Schematic representation of the antlion pit showing the optimal sand-throwing position at 0 deg, the vibrating rod emitting stimuli placed at 180 deg and an example of the larva's initial and final orientation. The angular displacement α represents the angle between the larva's starting position and the optimal launch direction.

At the beginning of each trial, the vibrating PVC bristles were put in contact with the sand at the edge of the pit, at a random location across its circumference (Fig. 1). The bristles were then moved towards and away from the centre of the pit, so that the tip of the PVC bristles slid along the radius of the pit, ensuring the maintenance of a fixed angular position relative to the centre, for a total duration of 1 min (see Movie 1). This was done in order to simulate a prey falling down and climbing the pit walls again, as this situation often triggers sand tossing in the natural environment. Because the antlion remained buried beneath the sand, its initial body orientation could not be determined visually at the trial onset. Therefore, larval orientation and position were identified *a posteriori* using the video recordings. In fact, the antlion became visible upon the first sand-tossing event, which allowed us to detect its initial visible orientation. The same was done for all the other positions, orientations and directions of movement (clockwise or counterclockwise) that antlions occupied across the duration of the trial. At the end of the trial, a single ant was dropped in the pit as food reward, to ensure the animal’s survival and to maintain motivation. Then, the experiment proceeded with the next animal. All trials were conducted between 10:00 h and 17:00 h. Each antlion was tested a maximum of 3 times in a day, with at least 1 h between each trial.

### Behavioural scoring and angular measurements

A total of 128 trials were performed (see Table S1 for a detailed list of trial dates). The antlions produced a response in 45 of them (see Table S2 for the outcome of each trial), corresponding to 35.15%. If during the stimulation the antlion did not produce any response, the video was excluded from the analysis. For each responding trial, larvae behaviour was analysed offline from video recordings using the software BORIS (Behavioral Observation Research Interactive Software) (Friard and Gamba, 2016). To allow comparison across trials, a common angular reference frame was defined *post hoc* by arbitrarily assigning the angular position of the stimulus (i.e. the vibrator bristles) as 180 deg (Fig. 1).

Given the low visibility of the antlion under the sand, it was difficult to pinpoint the exact orientation and we could only determine it roughly. Accordingly, all angular positions were approximated to the nearest 45 deg, resulting in eight possible initial orientation categories (0, 45, 90, 135, 180, −135, −90, −45 deg, where negative numbers represent counterclockwise orientation). Thereafter, the video was observed to determine the rotational direction of the antlion, be it clockwise or counterclockwise (Fig. 1). Finally, we also recorded the orientation of the antlion by the end of the trial and set this as the final orientation. Larval movement and direction of rotation (clockwise or counterclockwise) were then tracked throughout the video of each trial, while the final orientation of the antlion was defined as the position of the larva at the end of the 1 min stimulation period.

### Statistical analysis

Using the initial and final orientation of the animal along with the rotation direction, we calculated the total rotated angle α (again expressed as negative numbers for counterclockwise and positive numbers for clockwise rotations). The calculations were done in Python 3 (Van Rossum and Drake, 1995), using the packages Pandas (available from Zenodo: doi:10.5281/zenodo.3509134) and Numpy (Harris et al., 2020).

Statistical analysis was performed in R (https://www.r-project.org/). To start, we binned the observed initial orientation of the antlions in four separate groups: (1) ‘positive’ (i.e. with angles between 45 deg and 135 deg with respect to the optimal sand-tossing orientation) – in this case, we expected the antlions to rotate counterclockwise, being the shortest rotation direction to reach 0 deg; (2) ‘negative’ (i.e. with angles between −45 deg and −135 deg with respect to the optimal sand-tossing orientation) – for these, we instead expected clockwise rotations; (3) optimal (i.e. already at the optimal sand-tossing orientation, 0 deg) – for these, we expected the antlions to not rotate at all; and (4) reversed (i.e. exactly opposite to the optimal orientation, 180 deg) – for these trials, we had no *a priori* expectation, as rotating clockwise or counterclockwise was identical in terms of distance to reach 0 deg.

We then tested the probability of antlions rotating clockwise, counterclockwise or not rotating, depending on initial orientation, using χ^2^ analysis. We then performed a Bonferroni-corrected *post hoc* test using the package chisq.posthoc.test (Beasley and Schumacker, 1995).

## RESULTS AND DISCUSSION

Raw data and analysis are publicly available from Zenodo (doi:10.5281/zenodo.19063729). Of the 45 recorded trials, 12 had the antlion already perfectly oriented (0 deg) with respect to the artificial prey, while 5 had it oriented oppositely (180 deg). Of the remaining 28, in 12 the antlions were initially positioned at positive angles and in the other 16 they were initially positioned at negative angles (Table 1).

**Table 1.** Summary of antlion rotations according to initial orientation relative to the vibrational stimulus

Antlion starting orientation	Rotated clockwise	Rotated counterclockwise	No rotation
Optimal (0 deg)	0	1	11
Positive angles (45 to 135 deg)	2	9	1
Negative angles (–45 to –135 deg)	10	1	5
Reverse orientation (180 deg)	3	1	1

The number of responding trials is grouped by initial orientation position as ‘optimal’ (0 deg), ‘positive angles’ (45 to 135 deg), ‘negative angles’ (−45 to −135 deg) or ‘reverse orientation’ (180 deg). The table also reports the number of trials in which no reorientation was observed.

Overall, 41 antlions ended the trial oriented in an advantageous throwing position, between +45 deg and −45 deg, equalling more than 90% of the trials (28 finished at 0 deg). In the 4 trials where subjects did not finish the trial in an optimal orientation, they did not reorient at all. The contingency table (Table 1) was overall statistically different from chance (χ^2^=36.582, d.f.=6, *P*<0.0001).

**Fig. 2.**
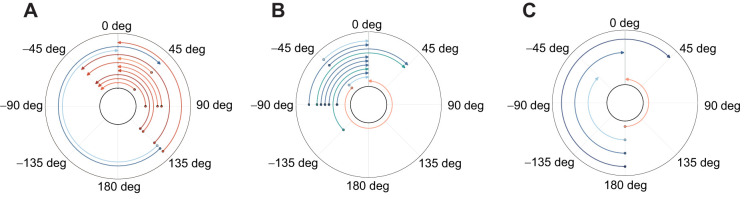
**Polar plot describing antlion rotation during the trial.** Plots are shown for antlions that started from positive angles (A), antlions that started from negative angles (B) and those with reversed orientation (C). Trials with the antlions starting from optimal positioning are not shown. Each arrow represents a single trial indicating rotation direction and distance: red arrows correspond to counterclockwise rotations, blue arrows to clockwise rotations, with colour intensity proportional to the angular displacement. Antlions that did not rotate in the trial are not shown in the plot. 0 deg indicates the optimal launch orientation; 180 deg indicates the reverse orientation.

The *post hoc* test revealed that in trials where antlions started at negative orientations, there was a higher-than-chance frequency of clockwise rotation (residuals=3.083, *P*=0.0246), while the frequency of counterclockwise rotation (residuals=−2.3, *P*=0.257) and no rotation (residuals=−0.89, *P*=1) was not different from chance. For trials where the antlions started instead at positive orientations, we observed a significantly higher-than-chance frequency of counterclockwise rotation (residuals=4.421, *P*=0.0001) while the frequency of clockwise rotation (residuals=−1.43, *P*=1) and no rotation (residuals=−2.615, *P*=0.107) was not different from chance. Both of these met our hypothesis, as the shortest rotation direction to reach optimal was taken in both cases with a higher frequency than chance. Indeed, looking at the individuals' behaviour, only 1 out of 16 animals positioned at a negative angle rotated counterclockwise (while 5 did not rotate at all), and only 2 out of the 12 positioned at a positive angle rotated clockwise (while only 1 did not rotate; Fig. 2).

In trials where the antlions started as optimally oriented, we observed a higher-than-chance frequency of no rotation (residuals=4.266, *P*=0.0002), while the frequency of clockwise (residuals=−2.86, *P*=0.051) and counterclockwise (residuals=−1.677, *P*=1) rotations was not different from chance. This was in accordance with our expectation, as antlions that are already in the advantageous orientation should not rotate. For the trials where the antlions started oriented in reverse to the optimal position instead, we observed that the frequency of clockwise rotation (residuals=1.342, *P*=1), counterclockwise rotation (residuals=−0.357, *P*=1) or no rotation (residuals=−0.968, *P*=1) was not different from chance. Indeed, of the 5 trials where animals started at 180 deg, opposite to optimal orientation, 1 did not reorient, 3 rotated clockwise and 1 rotated counterclockwise. Even given this apparent imbalance, the sample is too small to suppose a rotational preference in the absence of an optimal choice.

Our results show that antlion larvae reorient toward prey-like vibrational stimuli in a non-random manner: rather than rotating indiscriminately or following a fixed directional bias, in responding trials larval turning direction depended on the initial angular position relative to the stimulus, such that subjects followed the shortest rotational path to reach an optimal sand-tossing orientation.

Among responding trials, individuals starting on the negative side of the stimulus axis rotated mainly clockwise, whereas those starting on the positive side rotated predominantly counterclockwise, indicating that initial orientation strongly influences turning direction. Although this pattern was strong overall, some trials deviated from the expected direction, highlighting that reorientation is not expressed uniformly across individuals and conditions.

This relationship suggests that larvae integrate spatial information from vibrational cues with a rule of angular movement minimisation. Furthermore, larvae positioned exactly at 180 deg, where the angular displacement required to rotate clockwise or counterclockwise is equal, showed a slight preference for counterclockwise rotation: however, the small sample size (*n*=5) prevents any strong inference about a directional preference in this specific condition.

An interesting observation emerging from our experiment is that larvae could initiate sand tossing even before achieving an optimal sand-throwing position. We know that the trigger for initiating sand throwing is the detection of mechanical vibrations (Mencinger-Vračko and Devetak, 2008; Büsse et al., 2021; Devetak et al., 2007a; Martinez et al., 2023): antlions can react immediately upon detecting prey-like cues but, if they find themselves misaligned with respect to the prey, they can subsequently adjust by rotating toward the appropriate orientation. We hypothesize that this mismatch between stimulus location and throwing direction might serve as the trigger for reorientation. Thus, the sequence may involve two distinct steps: an immediate vibrationally driven response, followed by a secondary, potentially error-driven correction when the first tosses prove ineffective. Although this remains speculative, it offers a plausible mechanistic framework for the observed behavioural pattern.

Notably, sand tossing was not observed in the majority of trials performed, with larvae reacting in only 35.15% of the cases. This may be due to the imperfect mimicry of our artificial vibratory stimulus, which possibly failed on some occasions in producing a similar enough simulation of a real prey. Because the vibratory device did not allow precise control over the frequency or amplitude of the stimulus, we cannot exclude that different amplitudes or frequencies might influence the elicitation of a behavioural response. However, for antlions that reacted to the stimulus, the subsequent behaviour is unlikely to be influenced by the vibration characteristic, instead reflecting the spatial relationship between the stimulus and the antlion's body orientation.

It is also possible that changing body orientation may be costly for pit-building antlions and therefore is not expected to occur often. In many trials, antlions faced with a vibratory stimulus coming from a disadvantageous throwing position may end up not responding at all, waiting for the prey to naturally move to a better location. In line with this interpretation, Farji-Brener et al. (2025) reported that antlion larvae often maintain a stable ambush orientation once established at the bottom of the pit because repositioning may compromise pit structure and reduce trap performance. Thus, reorientation in our experiment may represent a context-dependent adjustment that is expressed only when the expected benefit outweighs the cost of movement.

The use of substrate-borne vibrations to detect and localise prey is widespread among sit-and-wait arthropod predators (Virant-Doberlet et al., 2023; Strauß and Stritih-Peljhan, 2022), although sensory strategies and transmission media can differ across taxa. For instance, sand scorpions accurately estimate both direction and distance of a prey in dry sand when it is <15 cm away (Brownell and Farley, 1979a,b), perceiving vibrations produced by prey footsteps on the surface, thanks to the extremely sensitive mechanoreceptors for surface waves located at the tip of each walking leg (Brownell and Farley, 1979c; Brownell, 1977). Similar to the mechanism proposed for antlion larvae, sand scorpions infer prey direction by comparing wave arrival-time difference across their eight legs (Brownell and Farley, 1979b; Brownell and van Hemmen, 2001).

In web-building spiders, instead, prey localisation relies on the detection of vibrations transmitted through the web and the comparison of amplitude signals received by sensory organs located on the legs (Wu et al., 2023). In orb webs, however, the time-of-arrival differences of vibrations at the spider's legs are extremely small because of the high propagation velocity of web vibrations, which may limit their usefulness as directional cues (Landolfa and Barth, 1996).

In sensorimotor terms, antlion reorientation behaviour has a notable difference from that of other sit-and-wait predators. In scorpions or web-building spiders, vibrational cues typically trigger movement towards the prey source. In contrast, antlion larvae because of their sand-tossing behaviour constraints must rotate such that the stimulus ends up behind them. To achieve this, they often have to briefly move away from the prey, rather than towards it. This requires some level of basic planning in computation that disregards medial positions in order to reach the final optimal orientation.

In this paper, we have described how the antlion larvae implement an efficient strategy: when reorientation is required, they do not rotate indiscriminately but instead follow a minimisation rule to reach an optimal sand-tossing orientation, allowing them to maximise efficiency while minimising energy expenditure (Caraco and Gillespie, 1986).

## Supplementary Material

10.1242/jexbio.252138_sup1 Supplementary information
