# Supplementary material for: Antlion larvae follow optimality rules in body orientation during sand tossing
Source: J Exp Biol. 2026 May 13;229(9):jeb252138. doi: 10.1242/jeb.252138 (PMC13245912; doi:10.1242/jeb.252138)
Supplement: Supplementary information [file jexbio-229-252138-s1.pdf]

Table S1. Schedule of trials performed for each antlion larva (subjects OT001–OT019).
For each individual, the table reports the calendar date (day/month) on which each trial was conducted. Empty cells indicate that no further trials were performed for that subject.

Trial dates

(dd/mm)

Subject	Trial number 1	2	3	4	5	6	7	8	9	10	11	12
**OT001**	28/8	2/9	4/9	6/9	11/9	23/9	25/9					
**OT002**	28/8	2/9	4/9	6/9	11/9	23/9						
**OT003**	28/8	2/9	6/9	11/9	23/9							
**OT004**	28/8	2/9	4/9	4/9	6/9	6/9	11/9	23/9	7/10	7/11		
**OT005**												
**OT006**	28/8	2/9	4/9	4/9	4/9	6/9	6/9	11/9	23/9	25/9	7/11	
**OT007**	28/8	30/8	30/8	2/9	2/9	4/9	6/9	11/9	23/9	25/9		
**OT008**	28/8	30/8	30/8	2/9	2/9	4/9	4/9	6/9	11/9	23/9	25/9	7/11
**OT009**	28/8	30/8	2/9	2/9	4/9	6/9	11/9	23/9	25/9	7/11	7/11	
**OT010**	28/8	2/9	4/9	6/9	11/9	23/9	7/11	7/11				
**OT011**	2/9											
**OT012**	28/8	2/9	4/9	4/9	4/9	6/9	6/9	11/9				
**OT013**	28/8	2/9	4/9	6/9	11/9							
**OT014**	28/8	2/9	4/9	4/9	4/9	6/9	11/9	23/9	7/11			
**OT015**	28/8	2/9	4/9	6/9	6/9	11/9						
**OT016**												
**OT017**	2/9	4/9	4/9	4/9	6/9	11/9	23/9	25/9	7/11			
**OT018**	4/9	6/9	6/9	11/9	23/9	25/9	7/11					
**OT019**	11/9	23/9	7/11									

Table S2. Binary outcome of each trial for each antlion larva (subjects OT001–OT019).
Each cell indicates whether a detectable behavioural response to the vibrational stimulus was observed during the 1-min stimulation period (1 = response; 0 = no response). Empty cells indicate that no further trials were performed for that subject.

Trial outcomes

response/unresponse

Subject \ Trial number	1	2	3	4	5	6	7	8	9	10	11	12
**OT001**	0	0	0	0	0	1	0					
**OT002**	0	0	0	1	0	0						
**OT003**	0	0	1	0	0							
**OT004**	0	1	1	1	1	1	0	0	0	0		
**OT005**												
**OT006**	1	0	1	1	0	1	1	1	1	0	0	
**OT007**	1	0	0	0	0	0	0	1	0	0		
**OT008**	1	1	1	0	1	1	1	0	1	0	0	1
**OT009**	0	0	0	0	0	0	1	0	0	1	1	
**OT010**	0	1	0	0	1	0	0	0				
**OT011**	0											
**OT012**	0	0	0	1	1	1	1	0				
**OT013**	0	0	0	0	0							
**OT014**	0	0	1	1	0	1	0	0	1			
**OT015**	0	0	1	1	0	0						
**OT016**												
**OT017**	0	1	1	0	0	0	0	0	0			
**OT018**	1	1	1	0	0	0	0					
**OT019**	0	0	0									

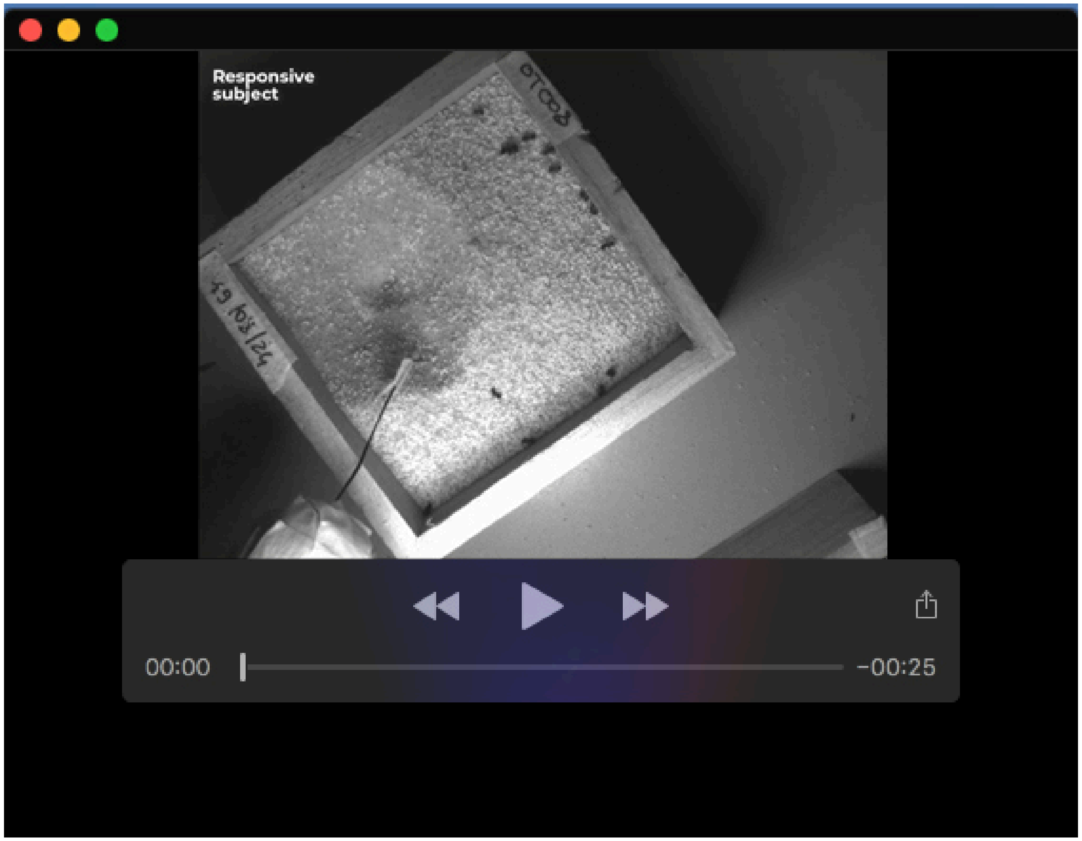

Movie 1. Representative video of a trial. In the first part, a larva responding to the stimulus is shown; in the second part, a larva not responding is shown.

Journal of Experimental Biology • Supplementary information
